# Clinical Characteristics and Short-Term Prognosis of Children With Antibody-Mediated Autoimmune Encephalitis: A Single-Center Cohort Study

**DOI:** 10.3389/fped.2022.880693

**Published:** 2022-07-08

**Authors:** Qingyun Kang, Hongmei Liao, Liming Yang, Hongjun Fang, Wenjing Hu, Liwen Wu

**Affiliations:** Department of Neurology, Hunan Children's Hospital, Changsha, China

**Keywords:** autoimmune encephalitis, child, clinical characteristics, immunotherapy, prognosis

## Abstract

**Background:**

The incidence and prevalence of autoimmune encephalitis (AE) is gradually increasing in pediatric patients (between the ages of 3 months and 16 years). The aim of this retrospective observational study was to investigate the clinical characteristics and short-term prognosis of children with antibody-mediated AE at Hunan Children's Hospital.

**Methods:**

Antibody analysis of blood and/or cerebrospinal fluid was performed in suspected AE patients admitted to the Department of Neurology, Hunan Children's Hospital from June 2014 to June 2021. Ultimately, 103 patients were diagnosed with antibody-mediated AE and were enrolled in this study. Clinical data and corresponding demographic, clinical characteristics, laboratory and imaging data, treatment, and prognosis data were collected and analyzed.

**Results:**

In our study, 103 AE patients with antibody-positive were identified. The main subtype of AE in our cohort was anti-NMDAR encephalitis. Few patients have anti-CASPR2 encephalitis, anti-GABABR encephalitis, or anti-LGI1 encephalitis. In our AE patients, the most common clinical manifestations were behavioral symptoms, seizures, and involuntary movements, with seizures being the most common initial symptom. All patients underwent brain magnetic resonance imaging (MRI) and electroencephalography (EEG). Forty-five (43.7%) patients had abnormal MRI findings. And 96 (93.2%) patients had abnormal EEG results. All 103 patients were given first-line immunotherapy, 21 of which were also treated with the combination of the second-line immunotherapy. All surviving patients were followed up for at least 6 months. Seventy-seven patients recovered completely, 23 had sequelae of different degrees, and 3 died. Eight patients had one or more relapses during the follow-up period.

**Conclusions:**

AE is a treatable disease that can occur in children of all ages. The mortality rate is low, as most patients have a good response to immune therapy. Compared with the older children, infants and young children (≤ 3 years old) with anti-NMDAR encephalitis have a higher incidence of fever and status epilepticus, more severe condition, higher PICU admission rate and worse prognosis. AE patients with high maximum mRS scores and PICU admissions may require second-line immunotherapy.

## Introduction

Autoimmune encephalitis (AE) is a group of encephalitides caused by autoimmune mechanisms mediated by immune responses against the central nervous system (CNS) antigens. The disease spectrum of AE has been expanding since the first case of the anti-N-methyl-D-aspartic acid receptor (NMDAR) encephalitis was identified (1). The discovery and study of new anti-neural antibodies have made great strides in the study of various aspects of the etiology, pathogenesis, and treatment of AE (2–4).

AE affects the quality of patients' life and imposes a serious economic burden on patients and society (5). However, most patients with autoimmune encephalitis are sensitive to immunotherapy. Previous studies have shown that early diagnosis and timely immunotherapy are key to improving the prognosis of AE (6, 7). Increased awareness of autoimmune encephalitis, early recognition, and timely completion of relevant antibody tests can improve the diagnosis rate of the disease. Although there are many studies on the clinical presentation, laboratory findings, immunotherapy, short-term prognosis, and other clinical data of AE (8–16), most studies have focused on adults. Studies on pediatric patients are still limited. More studies are needed for a better understanding of AE in children.

Here, we retrospectively analyzed the clinical characteristics, immunotherapy and short-term prognosis of children with AE admitted to Hunan Children's Hospital, to provide a reference for the diagnosis and treatment of autoimmune encephalitis in children.

## Materials and Methods

### Participants

The study was approved by the Ethics Committee of Hunan Children's Hospital. In this retrospective cohort study, a total of 106 patients with positive neuron surface antibodies were identified by antibody testing. Three antibody-positive patients were excluded from further clinical analysis because of alternative diagnoses. Two patients with positive anti-CASPR2 antibodies detected in sera but not in CSF were eventually diagnosed with tuberous sclerosis and urea cycle disorder, respectively. One patient with positive anti-NMDAR antibodies detected in sera but not in CSF was eventually diagnosed with hereditary epilepsy. The other 103 patients who met the criteria for the AE criteria suggested by Graus et al. (8) were ultimately enrolled in our study: (1) subacute onset (rapid progression <3 months) with working memory deficits, altered state of consciousness, or behavioral symptoms; (2) presence of at least one of the following: new focal central nervous system (CNS) findings, seizures unexplained by previously known seizure disorders, CSF pleocytosis (white blood cell count >5 cells/mm^3^), or MRI features suggestive of encephalitis; and (3) reasonable exclusion of alternative causes. All subjects in the study were free of any disability prior to the onset of the disease.

### Antibody Testing

The antibody panel included anti-NMDAR, anti-GABABR, anti-LGI1, anti-CASPR2, anti-AMPA1 receptor, and anti-AMPA2 receptor. The patients' blood and cerebrospinal fluid samples were sent to Wuhan Kindstar Medical Laboratory and Guangzhou King Med Center for Clinical Laboratory. The laboratory analyzed the cerebrospinal fluid and serum of each patient using a highly specific and sensitive cell-based assay (CBA). The initial dilution titers of CSF and serum were 1:1 and 1:10, respectively.

### Clinical Data Analysis

Clinical data of all subjects were retrospectively analyzed, including demographic characteristics, clinical symptoms, MRI findings, video EEG monitoring, cerebrospinal fluid findings, serum tumor markers, chest CT scan or x-ray, abdomen, and pelvic cavity CT scans or ultrasound, treatment and prognosis. All patients were followed up every 3 months in the first year after discharge, and every 6 months since the second year. All patients were followed up for at least 6 months. We used the modified Rankin scale (mRS) to measure neurological outcomes in all AE patients. MRS scores were determined by two experienced doctors at the onset, at the worst condition (recorded as the maximum mRS score), and at the last visit (recorded as the terminal mRS score). An mRS score of 0 at the last visit was considered a good outcome and ≥1 a poor outcome. AE patients with mRS scores of 0 at the last visit after treatments were assessed as recovered completely. AE relapse was defined as a recurrence or worsening of symptoms at least 2 months after improvement or stabilization of AE.

### Statistical Analysis

SPSS 25.0 was used for statistical analysis. Measurements conforming to a normal distribution were presented as mean ± standard deviation. And two independent samples *t*-test was used for comparisons between groups. Statistical data were presented as percentages, and comparisons between groups were made using the χ^2^ test or Fisher's exact probability method. *P* < 0.05 was considered a statistically significant difference.

## Results

### Demographic Features of AE Patients

**Figure 1:**
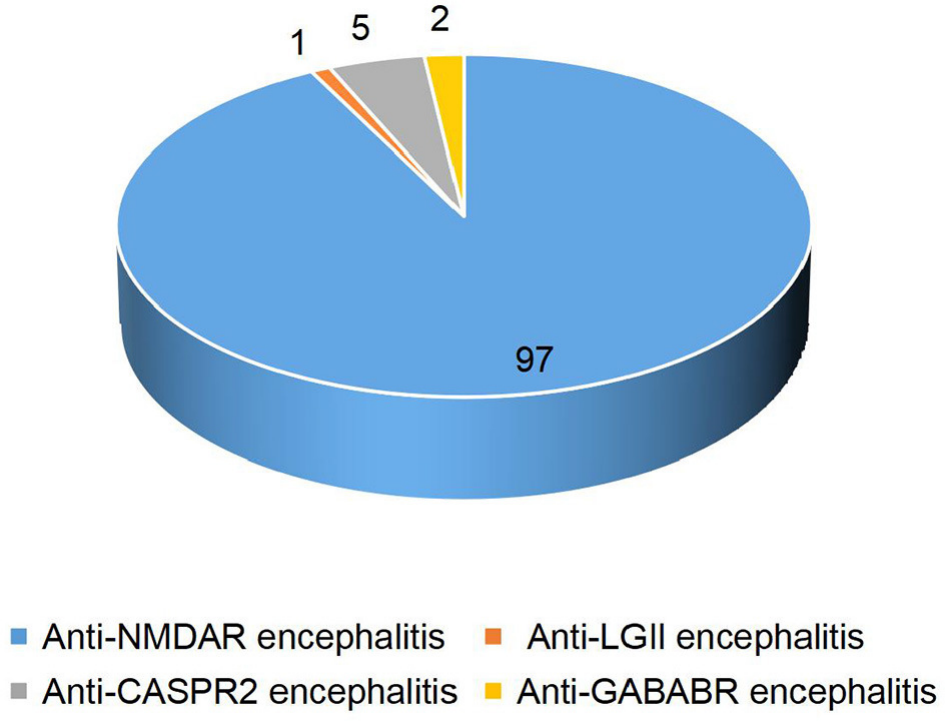
Autoimmune encephalitis classification in children.

**Figure 2:**
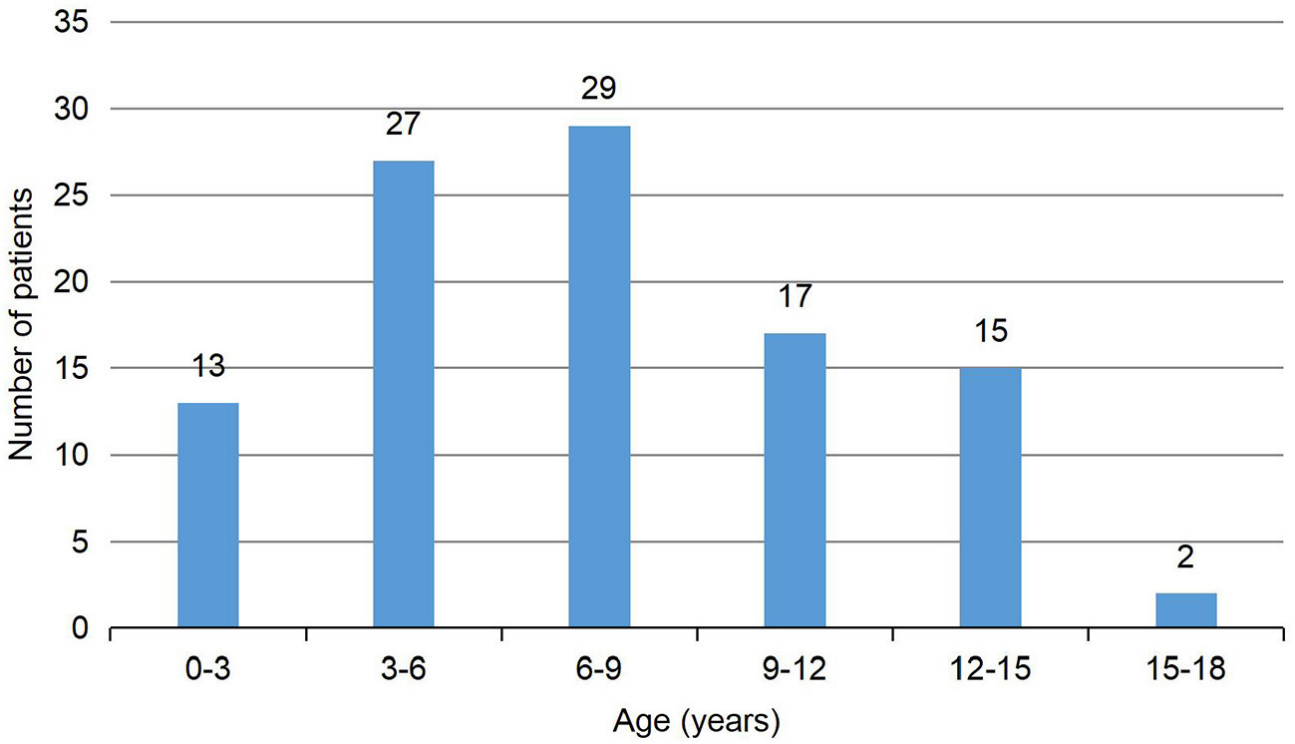
Distribution of age of patients with autoimmune encephalitis.

In our study, detailed clinical data of 103 patients with antibody-positive autoimmune encephalitis were collected. Among these cases, 97 patients had anti-NMDAR encephalitis (1 patient was positive for both anti-NMDAR and anti-CASPR2 antibodies), 5 patients had anti-CASPR2 encephalitis (1 patient was positive for both anti-CASPR2 and anti-GABABR antibodies), 2 patients had anti-GABABR encephalitis, and 1 patient had anti-LGI1 encephalitis (Figure 1). In patients with anti-NMDAR encephalitis, antibodies were detected in both serum and CSF in 69 patients, in CSF only in 19 patients, and in serum only in 9 patients. In the group of patients with anti-CASPR2 encephalitis, antibodies were detected in both serum and CSF in 2 patients and serum only in the other 3 patients. In the group of patients with anti-GABABR encephalitis, antibodies were detected in both serum and CSF in 1 patient, and in serum only in the other patient. In the group of 1 patient with anti-LGI1 encephalitis, autoantibodies were detected in both serum and CSF (Table 1). Of the 103 patients included in the study, 47 were males and 56 were females. The youngest patient was only 3 months old, and the oldest patient was 16 years old. The median age of AE onset in the included patients was 87.2 months, and the peak frequencies of AE onset occured at ages 3–6 and 6–9 years (Figure 2).

**Table 1:** Demographic data and clinical features in AE patients.

	**Patients**				
	**Autoimmune encephalitis** **(*n* = 103)**	**Anti-NMDAR** **(*n* = 97)**	**Anti-CASPR2** **(*n* = 5)**	**Anti-LGI1** **(*n* = 1)**	**Anti-GABABR** **(*n* = 2)**
**Demographic data**					
Age of onset range (month)	3–192	3–192	25–118	157	88–118
Sex (M:F)	47:56	42:55	4:1	0:1	2:0
**Types of onsets**, ***n*** **(%)**					
Acute (≤ 2 W)	63 (61.2%)	60 (61.9%)	2 (40%)	1 (100%)	1 (50%)
Sub-acute	24 (23.3%)	21 (21.6%)	3 (60%)	0	1 (50%)
Chronic (≥1 M)	16 (15.5%)	16 (16.5%)	0	0	0
**Positive antibody**, ***n*** **(%)**					
CSF-serum pairs	73 (70.9%)	69 (71.1%)	2 (40%)	1 (100%)	1 (50%)
Only CSF	19 (18.4%)	19 (19.6%)	–	–	–
Only sera	11 (10.7%)	9 (9.3%)	3 (60%)	–	1 (50%)
**Initial symptoms**, ***n*** **(%)**					
Seizures	52 (50.5%)	50 (51.5%)	2 (40%)	0	1 (50%)
behavioral symptoms	25 (24.3%)	22 (22.7%)	2 (40%)	1 (100%)	1 (50%)
Others	26 (25.2%)	25 (25.8%)	1 (20%)	0	0
**Clinical symptoms**, ***n*** **(%)**					
Seizures	68 (66.1%)	64 (65.9%)	3 (60%)	1 (100%)	2 (100%)
Status epilepticus	27 (26.2%)	25 (25.8%)	1 (20%)	1 (100%)	1 (50%)
behavioral symptoms	86 (83.5%)	81 (83.5%)	5 (100%)	1 (100%)	1 (50%)
Disturbance of consciousness	55 (53.4%)	51 (52.6%)	3 (60%)	1 (100%)	2 (100%)
Memory deficit	45 (43.7%)	36 (37.1%)	2 (40%)	1 (100%)	2 (100%)
Speech disorders	65 (63.1%)	61 (62.9%)	2 (40%)	1 (100%)	1 (50%)
Sleep disorder	58 (56.3%)	53 (54.6%)	4 (80%)	1 (100%)	1 (50%)
Involuntary movements	65 (63.1%)	65 (67%)	1 (20%)	0	1 (50%)
Headache	33 (32.0%)	32 (32.98%)	0	1 (100%)	0
Fever	41 (39.8%)	39 (40.2%)	1 (20%)	1 (100%)	2 (100%)
Autonomic dysfunction	28 (27.2%)	25 (25.8%)	3 (60%)	0	1 (50%)
Faciobrachial dystonic seizures	1 (0.97%)	0	0	1 (100%)	0
Ataxia	18 (17.5%)	18 (18.6%)	0	0	0
Visual defect	9 (8.7%)	9 (9.3%)	0	0	0
Sensory disorder	9 (8.7%)	9 (9.3%)	0	0	0

*M, male; F, female; NMDAR, N-methyl D-aspartate receptor; CASPR2, contactin-associated protein-like; GABABR, γ-aminobutyric acid B receptor; LGI1, leucine-rich glioma inactivated 1; W, week; M month*.

### Clinical Features of AE Patients

Of the 103 patients with AE included in the study, 63 (61.2%) had an acute onset (≤ 2 weeks), 24 (23.3%) had a subacute onset (2 weeks to 1 month), and 16 (15.5%) had a chronic onset (≥1 month). In our AE patients, the most common initial symptom was seizures, with behavioral symptoms being the most common clinical manifestation in the course of the disease. Other common clinical symptoms included disturbance of consciousness, memory deficit, speech disorders, involuntary movements, sleep disorder, headache, fever, and autonomic dysfunction. Some patients can also present with sensory disorders, ataxia, and visual defect. Hyponatremia and faciobrachial dystonic seizures were observed in our patient with anti-LGI1 encephalitis. Clinical features of patients with different types of AE are summarized in Table 1.

### Auxiliary Examinations in AE Patients

**Figure 3:**
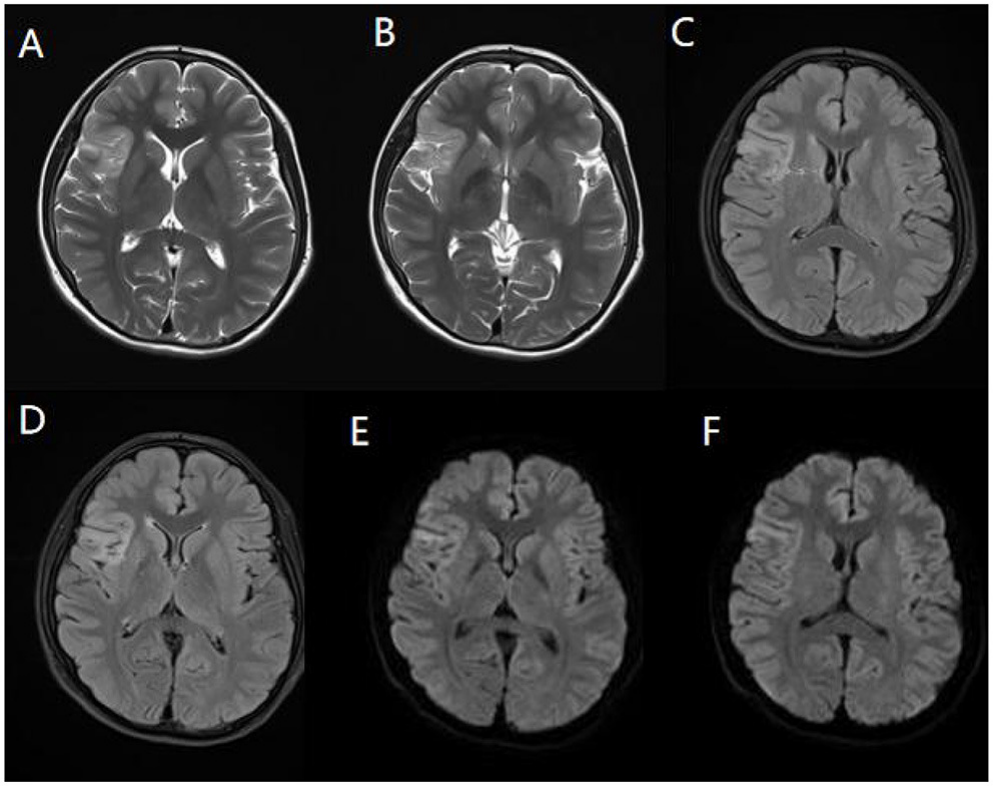
Magnetic resonance imaging (MRI) of a representative pediatric patient admitted for convulsions for 2 weeks and diagnosed with anti-NMDA receptor encephalitis (NMDA receptor antibody in CSF was 1:100). Right temporal and insula lobes and frontal cingulate gyrus showed lamellar abnormal signasl. T2WI showed a high signal **(A,B)**. FLAIR sequence was dominated by a high signal **(C,D)**, and DWI showed a high signal **(E,F)**.

**Figure 4:**
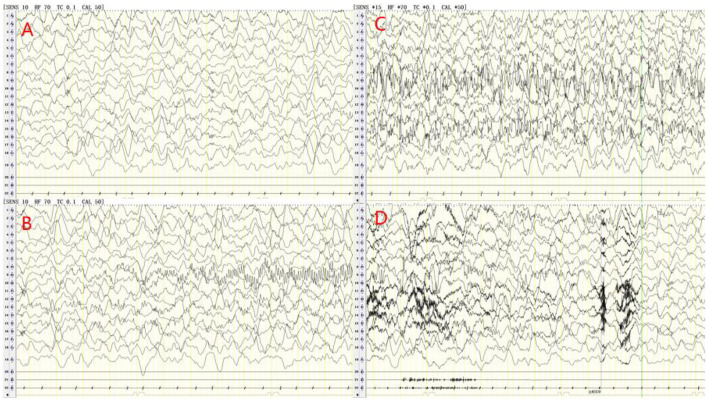
Electroencephalogram (EEG) of a 6-years old male patient with anti-NMDA receptor encephalitis. The delta rhythm (1.2–3 Hz, 80–250 UV) was used as the main background, with a small amount of θ rhythms (4–5 Hz, 40–100 UV) and slightly more low amplitude beta activity **(A)**. During video-EEG monitoring, the child had a seizure with oblique eyes, unconsciousness and smack mouth. The synchronous EEG was the mid-amplitude spike rhythm starting from the left occipital and posterior temporal regions, and the discharge stopped briefly after intravenous injection of midazolam **(B–D)**.

Cerebrospinal fluid (CSF) examination was performed in 102 of our 103 AE patients (prior to IVIG application). CSF pleocytosis (white blood cell count >5/mm^3^) was observed in 74 patients, and the maximum white blood cell count observed was 137/mm^3^. Elevated CSF protein levels (>500 mg/L) were observed in 9 patients, with the highest protein level being 820 mg/L. Brain MRI and EEG were performed and evaluated in all 103 patients. Brain MRI abnormalities were observed in 45 patients. Typical imaging findings of patients included in the study are shown in Figure 3. In the anti-NMDAR encephalitis group, abnormal brain MRI findings were observed in 43.3% of patients: abnormal fluid-attenuated inversion recovery (FLAIR) sequence signals associated with anti-NMDAR encephalitis were mainly found in the parietal, frontal, or temporal lobes. In the anti-CASPR2 encephalitis group, brain MRI abnormalities were observed in 2 patients (40%). All patients in the anti-GABABR and anti-LGI1 encephalitis groups had abnormal MRI findings in our study. EEG abnormalities were observed in 96 patients (93.2%). The most common abnormal EEG findings were focal or generalized non-specific slow waves (91.3%) and epileptiform discharges (such as sharp waves, spike waves, sharp slow wave complexes, or spike slow wave complexes) (41.7%).Twelve patients had seizures during video-EEG monitoring. A typical abnormal EEG showed diffusely slow waves and diffuse spike waves in Figure 4.

All 103 patients underwent chest X-rays or CT examinations. No tumors were found and only 5 patients were found to have pneumonia. We performed abdominal ultrasound or abdominal CT in 103 AE patients. Teratomas were observed in 2 patients with anti-NMDAR encephalitis, and the remaining patients had normal abdominal ultrasound or abdominal CT results.

In our study, 4 patients had anti-NMDAR encephalitis about 1–2 months following herpes simplex encephalitis (HSE). And HSE in these patients was confirmed by herpes simplex virus (HSV) PCR and HSV IgM antibody measurements of CSF. Multiple neuroautoantibodies were found to coexist in some patients. Six patients with anti-NMDAR and anti-MOG antibodies underwent MRI examinations of the spine and the optic nerve, of which 2 patients had abnormal spinal MRI and 4 patients had abnormal optic nerve MRI. Two patients had both anti-NMDAR antibodies and anti-GFAP antibodies. One patient had anti-NMDAR antibodies coexisting with anti-CASPR2 antibodies. And the other patient had anti-GABABR antibodies coexisting with anti-CASPR2 antibodies (Table 2).

**Table 2:** Auxiliary examinations of AE patients.

	**Patients**				
	**Autoimmune encephalitis** **(*n* = 103)**	**Anti-NMDAR ** **(*n* = 97)**	**Anti-CASPR2** **(*n* = 5)**	**Anti-LGI1** **(*n* = 1)**	**Anti-GABABR** **(*n* = 2)**
**CSF analysis**, ***n*** **(%)**					
Increased level of protein	9 (8.7%)	6 (6.2%)	1 (20%)	0	0
CSF pleocytosis (>5/mm^3^)	74 (71.8%)	69 (71.1%)	4 (80%)	1 (100%)	2 (100%)
**Patients with abnormal brain MRI findings**					
Total with abnormal findings	45 (43.7%)	42 (43.3%)	2 (40%)	1 (100%)	2 (100%)
Parietal lobe	29 (28.2%)	28 (28.9%)	1 (20%)	0	1 (50%)
Temporal lobe	23 (22.3%)	21 (21.6%)	1 (20%)	1 (100%)	1 (50%)
Frontal lobe	26 (25.2%)	25 (25.8%)	1 (20%)	0	1 (50%)
Occipital lobe	16 (15.5%)	15 (15.5%)	1 (20%)	0	1 (50%)
Thalamus	17 (16.5%)	15 (15.5%)	2 (40%)	0	2 (100%)
Insular lobe	10 (9.7%)	9 (9.3%)	1 (20%)	0	1 (50%)
Basal ganglia	8 (7.8%)	7 (7.2%)	0	0	1 (50%)
Cerebellum	6 (5.8%)	6 (6.2%)	0	0	0
Brain stem	2 (1.9%)	2 (2.1%)	0	0	0
Others	6 (5.8%)	5 (5.2%)	1 (20%)	0	1 (50%)
**Patients with abnormal EEG**					
Total with abnormal findings	96 (93.2%)	90 (92.8%)	5 (100%)	1 (100%)	2 (100%)
Slow waves	94 88.3 (%)	88 (90.7%)	5 100%)	1 (100%)	2 (100%)
Epileptic discharges	43 (41.7%)	39 (40.2%)	2 (40%)	1 (100%)	2 (100%)
**Others**					
Complicated with tumors	2 (1.9%)	2 (2.1%)	0	0	1 (50%)
MOG-positive (serum)	6 (5.8%)	6 (6.2%)	0	0	0
Secondary to HSV encephalitis	4 (3.9%)	4 (4.1%)	0	0	0

### Treatment and Outcomes

First-line immunotherapy (intravenous immunoglobulin, high-dose steroids, and plasma exchange) was used to care for pediatric patients with a diagnosis of AE. In our study, all 97 patients with anti-NMDAR encephalitis received the first-line immunotherapy. Two of these patients received IVIG only and 1 patient received a combination regimen of oral prednisone and IVIG. The remaining 94 patients were treated with a combination regimen of intravenous methylprednisolone (15–20 mg/kg/d for 5 days) and IVIG (400 mg/kg/d for 5 days). We used the modified Rankin scale (mRS) to assess the clinical efficacy on the 10–14th days of the treatment. Forty-two patients recovered well, 2 patients died due to autonomic dysfunction, and 53 patients recovered poorly. Of the 53 patients with poor recovery, 33 patients received a repeated regimen of intravenous methylprednisolone, with efficacy assessed weekly. A second round of intravenous methylprednisolone was determined based on clinical outcomes. Other 20 of 53 patients received the second-line immunotherapy (19 patients received rituximab and 1 patient received cyclophosphamide), and three of these patients were switched to the second-line immunotherapy after plasma exchange had failed (Table 3). Two patients with teratomas underwent tumor resection. As shown in Table 4, among patients with anti-NMDAR encephalitis, there was a statistical significance (*P* < 0.05) between the groups of repeat first-line immunotherapy and second-line immunotherapy in terms of admission to the pediatric intensive care unit (PICU) and maximum mRS score.

**Table 3:** Summary of treatment and outcome results of AE patients.

	**Patients**				
	**Autoimmune encephalitis** **(*n* = 103)**	**Anti-NMDAR** **(*n* = 97)**	**Anti-CASPR2** **(*n* = 5)**	**Anti-LGI1** **(*n* = 1)**	**Anti-GABABR** **(*n* = 2)**
**Treatment**, ***n*** **(%)**					
Only Steroids	0	0	0	0	0
Only IVIG	2 (1.9%)	2 (2.1%)	0	0	0
Steroids + IVIG	101 (98.1%)	95 (97.9%)	4 (80%)	1 (100%)	1 (50%)
Steroids + IVIG +PE	3 (2.9%)	3 (3.1%)	0	0	0
Second-line treatment	21 (20.4%)	20 (20.6%)	1 (20%)	0	1 (50%)
Cases requiring PICU admission	28 (27.2%)	26 (26.8%)	2 (40%)	0	2 (100%)
**Prognosis**, ***n*** **(%)**					
Complete recovery	77 (74.8%)	73 (75.6%)	4 (80%)	1 (100%)	1 (50%)
Epilepsy	7 (6.8%)	7 (7.2%)	0	0	0
Cognitive dysfunction	16 (15.5%)	15 (15.5%)	1 (20%)	0	0
Movement disorder	5 (4.9%)	5 (5.2%)	0	0	0
Relapse	8 (7.8%)	8 (8.2%)	0	0	0
Mortality	3 (2.9%)	2 (2.1%)	0	0	1 (50%)

**Table 4:** The comparison of clinical data between groups of repeated first-line immunotherapy and second-line immunotherapy in patients with anti-NMDAR encephalitis, case (%).

**Clinical information**	**Total**	**Repeated first-line immunotherapy** **(*n* = 33)**	**Second-line immunotherapy (*n* = 20)**	**t/χ2**	***P***
Sex, male	20 (37.74%)	10 (30.30%)	10 (50.00%)	2.056	0.152
Age (month)	77.53 ± 46.8	76.58 ± 45.3	79.1 ± 50.34	−0.189	0.851
Average maximum mRS score	4.11 ± 0.67	3.88 ± 0.6	4.5 ± 0.61	−3.638	0.001
Average terminal mRS score	0.7 ± 1.31	0.58 ± 0.97^a^	0.9 ± 1.74^a^	−0.763	0.452
Pleocytosis	40 (75.47%)	23 (69.70%)	17 (85.00%)	/	0.325
Cranial MRI abnormalities	27 (50.94%)	16 (48.48%)	11 (55.00%)	0.212	0.646
EEG abnormalities	45 (84.91%)	26 (78.79%)	19 (95.00%)	/	0.234
Admission to PICU	18 (33.96%)	5 (15.15%)	13 (65.00%)	13.797	<0.001
Fever	23 (43.40%)	11 (33.33%)	12 (60.00%)	3.605	0.058
Seizures	34 (64.15%)	21 (63.64%)	13 (65.00%)	0.01	0.92
Status epilepticus	17 (32.08%)	12 (36.36%)	5 (25.00%)	0.738	0.39
behavioral symptoms	46 (86.79%)	28 (84.85%)	18 (90.00%)	/	0.697
Disturbance of consciousness	35 (66.04%)	20 (60.61%)	15 (75.00%)	1.15	0.283
Involuntary movements	42 (79.25%)	27 (81.82%)	15 (75.00%)	/	0.728
Positive for multiple antibodies	1 (1.89%)	1 (3.03%)	0 (0.00%)	/	>0.999
Prior diagnosis of HSE	2 (3.77%)	1 (3.03%)	1 (5.00%)	/	>0.999
with teratomas	1 (1.89%)	0 (0.00%)	1 (5.00%)	/	0.377

*/Fisher exact test without statistic; ^a^Compared with maximum mRS score, P < 0.05*.

**Figure 5:**
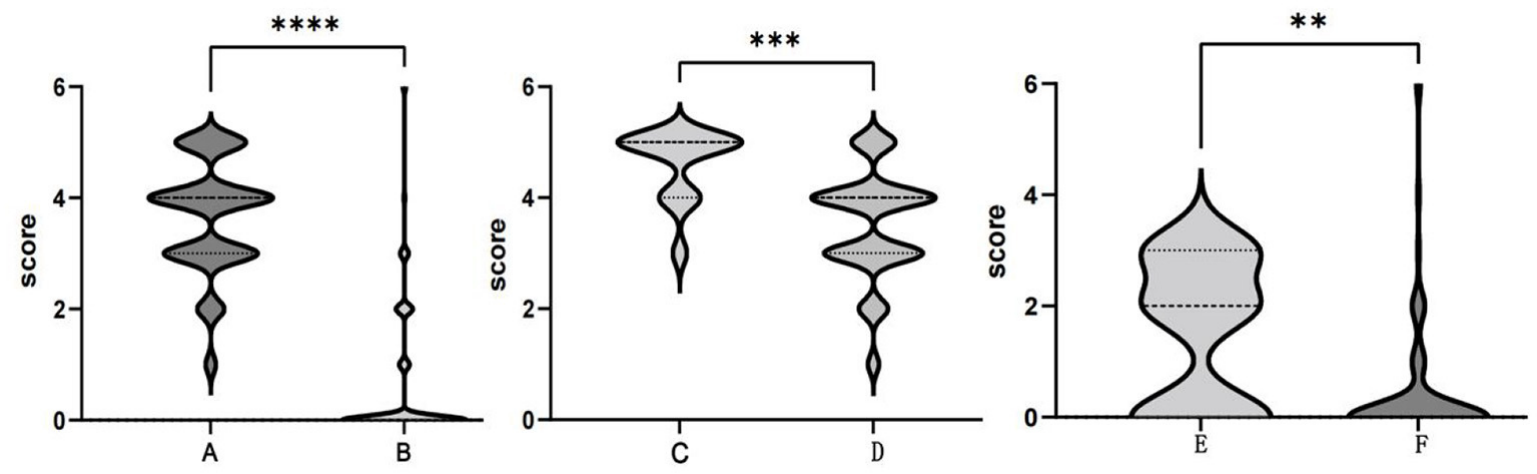
Comparison of the modified Rankin Scale (mRS) scores. The maximum mRS scores (A) and terminal mRS scores (B) in the anti-NMDAR encephalitis group. The maximum mRS scores in the ≤ 3- year-old group (C) and >3- year-old group (D). Terminal mRS scores in the ≤ 3- year-old group (E) and >3- year-old group (F). **P < 0.01; ***P < 0.001; ****P < 0.0001.

Patients with anti-NMDAR encephalitis were followed up for at least 6 months after discharge, with a median follow-up period of 39.4 months (ranging from 6.0 to 89.8 months). The mRS scores of patients at the last follow-up were significantly lower than those of patients at the worst status (Figure 5A,B). At the last follow-up, 73 patients (75.3%) recovered completely, and 2 patients (2.1%) died. Sixteen patients (16.5%) had mild disability, with an mRS score of 1 in 6 patients and 2 in 10 patients. Six patients (6.2%) had a severe disability, with an mRS score of 3 in 5 patients and 4 in 1 patient. Residual symptoms in patients with incomplete recovery included seizures, cognitive dysfunction, and motor impairment. Eight patients (8.2%) experienced one or more relapses during the follow-up period. Statistically significant differences (*P* < 0.05) could be identified between the completely recovered group and the incompletely recovered group in terms of maximum mRS scores. Patients in the incompletely recovered group had a higher incidence of fever and status epilepticus, higher PICU admission rate, and a higher number of HSE diagnoses preceding the diagnosis of AE (Table 5).

**Table 5:** Comparison of clinical data of patients with anti-NMDAR encephalitis between the completely-recovered and incompletely-recovered groups, case (%).

**Clinical information**	**Total**	**Incompletely-recovered** **(*n* = 24)**	**Completely-recovered** **(*n* = 73)**	**t/χ2**	***P***
Sex, male	42 (43.30%)	11 (45.83%)	31 (42.47%)	0.083	0.773
Age (month)	85.94 ± 45.5	73.92 ± 53.15	89.89 ± 42.36	−1.502	0.137
Average maximum mRS score	3.67 ± 1.01	4.29 ± 0.75	3.47 ± 1	3.708	<0.001
Pleocytosis	69 (71.13%)	18 (75.00%)	51 (69.86%)	0.232	0.63
Cranial MRI abnormalities	42 (43.30%)	13 (54.17%)	29 (39.73%)	1.534	0.215
EEG abnormalities	90 (92.78%)	24 (100.00%)	66 (90.41%)	/	0.188
Admission to PICU	26 (26.80%)	12 (50.00%)	14 (19.18%)	8.746	0.003
Fever	39 (40.21%)	14 (58.33%)	25 (34.25%)	4.359	0.037
Seizures	64 (65.98%)	18 (75.00%)	46 (63.01%)	1.156	0.282
Status epilepticus	25 (25.77%)	11 (45.83%)	14 (19.18%)	6.708	0.01
behavioral symptoms	81 (83.51%)	20 (83.33%)	61 (83.56%)	/	>0.999
Disturbance of consciousness	51 (52.58%)	15 (62.50%)	36 (49.32%)	1.259	0.262
Involuntary movements	65 (67.01%)	18 (75.00%)	47 (64.38%)	0.921	0.337
Positive for multiple antibodies	8 (8.25%)	1 (4.17%)	7 (9.59%)	/	0.675
Secondary to herpes simplex encephalitis	4 (4.12%)	3 (12.50%)	1 (1.37%)	/	0.046
with teratomas	2 (2.06%)	1 (4.17%)	1 (1.37%)	/	0.436

*/Fisher exact test without statistic*.

All 5 patients with anti-CASPR2 encephalitis were treated with a combination of intravenous methylprednisolone and IVIG, 1 of which was also treated with rituximab (14 days after the treatment of IVIG) because of poor efficacy. Four patients recovered completely and 1 patient had mild cognitive impairment.

Two patients had anti-GABABR encephalitis and were treated with a combination of intravenous methylprednisolone and IVIG. One of the patients was treated with rituximab (14 days after the treatment of IVIG) because of poor efficacy. One patient recovered completely after treatment and one patient died.

The only patient with anti-LGI1 encephalitis received a combination of intravenous methylprednisolone and IVIG and recovered after treatment.

### Young Children With Anti-NMDAR Encephalitis

Among our 97 patients with anti-NMDAR encephalitis, there were 13 infants and young children (≤ 3 years old), 7 of whom were admitted to the PICU, 9 had brain MRI abnormalities, and 9 had recurrent fevers and eight had status epilepticus during the disease period. The mean maximum mRS score and the mean terminal mRS score were 4.62 and 1.54, respectively. Eight patients had sequelae of different degrees, which includes 4 patients with mRS score of 2 and 4 patients with mRS score of 3. Compared with older children, infants and young children (≤ 3 years old) had a higher incidence of fever and status epilepticus, a higher cranial MRI abnormality rate, a higher PICU admission rate, a higher mean maximum mRS score (Figures 3C,D), a higher mean terminal mRS score (Figures 3E,F), and a lower incidence of behavioral symptoms. All of these differences were statistically significant (*P* < 0.05) (Table 6).

**Table 6:** Comparison of clinical data of patients with anti-NMDAR encephalitis, between the >3-year-old group and the ≤ 3-year-old group, case (%).

**Clinical information**	**Total**	**>3-year-old group (*n* = 84)**	**≤3-year-old group (*n* = 13)**	**t/χ2**	***P***
Sex, Male	42 (43.30%)	36 (42.86%)	6 (46.15%)	0.05	0.823
Average maximum mRS score	3.67 ± 1.01	3.52 ± 0.98	4.62 ± 0.65	−3.894	<0.001
Average terminal mRS score	0.59 ± 1.23	0.44 ± 1.15^a^	1.54 ± 1.33^a^	−3.126	0.002
Pleocytosis	69 (71.13%)	61 (72.62%)	8 (61.54%)	/	0.512
Cranial MRI abnormalities	42 (43.30%)	33 (39.29%)	9 (69.23%)	4.112	0.043
EEG abnormalities	90 (92.78%)	77 (91.67%)	13 (100.00%)	/	0.589
Admission to PICU	26 (26.80%)	19 (22.62%)	7 (53.85%)	/	0.038
Fever	39 (40.21%)	30 (35.71%)	9 (69.23%)	5.26	0.022
Seizures	64 (65.98%)	52 (61.90%)	12 (92.31%)	/	0.055
Status epilepticus	25 (25.77%)	17 (20.24%)	8 (61.54%)	/	0.004
behavioral symptoms	81 (83.51%)	73 (86.90%)	8 (61.54%)	/	0.037
Disturbance of consciousness	51 (52.58%)	42 (50.00%)	9 (69.23%)	1.67	0.196
Involuntary movements	65 (67.01%)	54 (64.29%)	11 (84.62%)	/	0.209
Positive for multiple antibodies	9 (9.28%)	8 (9.52%)	1 (7.69%)	/	>0.999
Secondary to HSE	4 (4.12%)	3 (3.57%)	1 (7.69%)	/	0.443

*/Fisher exact test without statistic; ^a^Compared with maximum mRS score, P < 0.05*.

## Discussion

With the increased awareness of AE and the ongoing discovery of relevant autoantibodies, an increasing number of encephalitis with unclear etiology have been diagnosed as AE. However, most studies reported on autoimmune encephalitis have focused on adult patients, and relatively few studies have been conducted on pediatric patients (9–11).

Among our 103 pediatric AE patients, 97 patients had anti-NMDAR encephalitis. The anti-NMDAR encephalitis was the most common type of AE in children, which is consistent with the previous literature (11). Adult patients with anti-NMDAR encephalitis often have behavioral symptoms as the most common initial symptom. While seizures are often cited as the most common presenting symptom in children with anti-NMDA receptor encephalitis. In our study, the seizure was the initial symptom in 50 (51.5%) patients with anti-NMDAR encephalitis, which is similar to the previous report (12). In our patients, the most common clinical manifestations were behavioral symptoms, seizures, and involuntary movements. Other symptoms, such as disturbance of consciousness, speech disorders, status epilepticus, sleep disorder, and memory deficit, were as common as those reported in other literature. Infectious factors have been speculated to trigger a pro-inflammatory state. The immune system, including microglia and immune cells in the CNS, can be activated and then produce an autoimmune response against the CNS (13). This might explain the higher prevalence of fever and headache in anti-NMDAR encephalitis, which is consistent with our study and previous reports (14). In our study, the youngest age of onset in patients with anti-NMDAR encephalitis was 3 months, suggesting that the disease can occur at all stages of childhood. In the infant and young children (≤ 3 years old) group, the incidence of fever and status epilepticus during the disease was higher, the disease was more severe, and the average maximum mRS score was higher. The prognosis was worse in younger children than in older children. We speculate that this may be related to the high incidence of fever and status epilepticus in younger children, the occurrence of which may exacerbate brain damage. Such differences have not been reported in other reports, which need to be verified in studies with larger samples.

With the continuous expansion of the anti-neural antibody spectrum and the widespread application of antibody testing, more and more patients with AE have been diagnosed. Recently, an increasing number of patients with autoimmune encephalitis have been found to have overlapping autoantibodies. The overlap of antibodies, especially pathogenic antibodies, can lead to an overlap of clinical phenotypes. In our study, 6 patients with concomitant anti-NMDAR antibodies and anti-MOG antibodies presented with clinical symptoms of both acute demyelinating diseases and anti-NMDAR encephalitis. The coexistence of anti-NMDAR antibodies and anti-MOG antibodies has been reported in previous studies (17, 18). In addition, there is an overlap between anti-NMDAR antibodies and other neurological autoantibodies. The pathological immune mechanisms underlying the phenomenon of multiple antibody overlap are unknown. It is speculated that simultaneous or successive exposure to different autoantigens under the condition of abnormal immune activation may initiate a series of autoimmune reactions that produce different autoantibodies and induce different autoimmune diseases. The presence of multiple neurological autoantibodies in the same patient has caused great confusion in clinical diagnosis and treatment. Whether the coexistence of multiple neuroimmune diseases can be diagnosed based on the presence of multiple neurological autoantibodies and whether each antibody can be used as a basis for disease monitoring and efficacy determination remains to be explored and resolved.

In our study, 4 patients had HSV infection in the CNS, subsequently, these patients had NMDAR encephalitis about 1–2 months following HSE. HSE and other possible viral encephalitides can trigger antibodies against the NMDAR and other neuronal cell-surface proteins (19). The molecular mimicry or disruption of immune tolerance may be the causation (20). More and more studies have reported that some patients with viral encephalitis, especially those with herpes simplex encephalitis and Japanese encephalitis, have a typical course of “bimodal encephalitis,” with the first phase being viral encephalitis and the second phase being the propagation of auto-antibodies wrongly targeting otherwise healthy cells. When patients recovering from viral encephalitis show symptoms such as behavioral symptoms, memory deficit, involuntary movements, and autonomic dysfunction, AE should be considered as a possibility and treated as early as possible.

In a study reported by Zhang et al. 89 pediatric patients had anti-NMDAR encephalitis (11). Among these patients, 29 patients (32.6%) had cranial MRI abnormalities, which were most commonly located in the temporal lobe (23.6%). This result was similar to our study (total abnormality in 43.3%, temporal lobe abnormality in 21.6%). This result might explain the high incidence of epileptic seizures in patients with AE, as medial temporal structures are key to the neural circuitry and play an important role in seizure propagation. In addition to frequent temporal lobe involvement, the frontal and parietal lobes were often involved in our cohort, with higher rates of frontal and parietal abnormalities than in previous studies (14). In our study, the rate of MRI abnormalities was higher in younger children than in older children, and this difference needs to be verified in a larger sample study. Consistent with previous studies, abnormal EEG signals were commonly observed in our study. Slow wave indicated impaired brain function, and epileptic discharge indicated clinical or subclinical epileptic activity (21, 22).

The treatment of patients with antibody-mediated autoimmune encephalitis mainly includes first- and second-line immunotherapy. A combination of steroids and IVIG was most frequently used in our cohort, and repeated intravenous methylprednisolone was used in some patients during the disease period. In contrast, second-line immunotherapy was applied to a relatively small portion of patients due to concerns about side effects and off-label use of RTX for AE in China. This situation is similar to previous reports in China (21, 23, 24). In Western countries, the second-line therapy is applied to a much larger proportion of patients, as it is commonly used in those who have failed the first-line therapy (12). However, in China, second-line immunotherapy is only used in a relatively small proportion of AE patients, usually in patients with severe or relapsing diseases. Our research suggests that patients with a higher maximum mRS score and PICU admission may not respond well to the first-line immunotherapy, and therefore may require the second-line immunotherapy. However, in the treatment of children with anti-NMDAR encephalitis, when aggressive immunotherapy is administered without significant improvement in clinical symptoms, whether to wait for natural improvement or to pursue more aggressive immunotherapy needs to be further validated in prospective large sample studies.

In our cohort, a high percentage of patients achieved a good prognosis, with 77 patients (74.8%) recovering completely. The rate of complete recovery was comparable to previous pediatric cohort studies (84.3%) (11). However, the rate of good prognosis in our cohort was lower than those in other study cohorts, because we used mRS score = 0 as a good prognosis criterion, whereas other studies considered mRS score ≤ 2 as a good prognosis (25). Data on morbidity and mortality in pediatric AE patients are less reported. Previous studies reported by Titulaer et al. and Zhang et al. showed low mortality rates in children with pediatric AE (2.7 and 1.1%, respectively), which were similar to our findings (mortality rate of 2.9%) (21, 26). The mortality rate of AE in pediatric patients is lower than that in adult patients. This may be due to the lower rate of tumors in children than in adults, children are less prone to autonomic dysfunction and hypoventilation. Of course, further statistical studies with more samples are needed to confirm these conjectures. Eight patients (7.8%) relapsed and improved after the second-line treatment, which was broadly consistent with the results of previous studies (11, 12).

In previous studies, patients who were treated early and did not require PICU admission during the disease had a good prognosis, whereas patients with a young age of onset, disturbance of consciousness, memory deficit, and status epilepticus had a poor prognosis (11, 12, 27, 28). In our study, admission to PICU and status epilepticus were high-risk factors for poor prognosis, which were consistent with previous findings. In contrast to previous studies, our study also found that recurrent fevers and secondary herpes simplex encephalitis were also high-risk factors for poor prognosis in patients with anti-NMDAR encephalitis.

Among all the AE patients in our study, only 2 patients with anti-NMDAR encephalitis had ovarian teratomas, suggesting that children, especially young children, rarely have tumors, which is consistent with the results reported in previous studies (11, 12). In addition, the proportion of our AE patients with fever, vomit, and headache was high, suggesting that infectious factors may be the major triggers for the development of AE in children.

Limitations of our study include its retrospective methodology, and the retrospective nature of the study, which may allow for selection bias. The mRS in real-time at the initial evaluations was documented, but there are retrospective data at the follow-up evaluations, which may result in inadequate accuracy during reporting. The inferences of this study are limited. In addition, the mRS score values for our good prognosis differed from previous studies, which could make the data less generalizable and/or ineligible for inclusion in a meta-analysis. However, this study is one of the largest series of pediatric autoimmune encephalitis patients in China, which has certain representativeness and provides a basis for future studies. In the future, prospective, large-scale, randomized, controlled trials can be conducted to further explore prognostic factors and validate the findings.

## Data Availability Statement

The original contributions presented in the study are included in the article/supplementary material, further inquiries can be directed to the corresponding author/s.

## Ethics Statement

Written informed consent was obtained from the minor(s)' legal guardian/next of kin for the publication of any potentially identifiable images or data included in this article.

## Author Contributions

QK conducted the literature review and drafted the manuscript. HL, LY, HF, and WH made substantial contributions to the conception and interpretation of data. LW was responsible for revising the manuscript critically and has given final approval for the version to be published. All authors contributed to the article and approved the submitted version.

## Funding

This work was supported by grants from the National Natural Science Foundation of China (No. 2021JJ30393).

## Conflict of Interest

The authors declare that the research was conducted in the absence of any commercial or financial relationships that could be construed as a potential conflict of interest.

## Publisher's Note

All claims expressed in this article are solely those of the authors and do not necessarily represent those of their affiliated organizations, or those of the publisher, the editors and the reviewers. Any product that may be evaluated in this article, or claim that may be made by its manufacturer, is not guaranteed or endorsed by the publisher.
